# Cause and Effectors: Whole-Genome Comparisons Reveal Shared but Rapidly Evolving Effector Sets among Host-Specific Plant-Castrating Fungi

**DOI:** 10.1128/mBio.02391-19

**Published:** 2019-11-05

**Authors:** William C. Beckerson, Ricardo C. Rodríguez de la Vega, Fanny E. Hartmann, Marine Duhamel, Tatiana Giraud, Michael H. Perlin

**Affiliations:** a Department of Biology, Program on Disease Evolution, University of Louisville, Louisville, Kentucky, USA; b Ecologie Systématique et Evolution, Université Paris-Sud, AgroParisTech, CNRS, Université Paris-Saclay, Orsay, France; University of California, Berkeley

**Keywords:** functional proteomics, effectors, small secreted proteins, host specificity, fungal pathogens

## Abstract

Plant pathogens utilize a portfolio of secreted effectors to successfully infect and manipulate their hosts. It is, however, still unclear whether changes in secretomes leading to host specialization involve mostly effector gene gains/losses or changes in their sequences. To test these hypotheses, we compared the secretomes of three host-specific castrating anther smut fungi (*Microbotryum*), two being sister species. To address within-species evolution, which might involve coevolution and local adaptation, we compared the secretomes of strains from differentiated populations. We experimentally validated a subset of signal peptides. Secretomes ranged from 321 to 445 predicted secreted proteins (SPs), including a few species-specific proteins (42 to 75), and limited copy number variation, i.e., little gene family expansion or reduction. Between 52% and 68% of the SPs did not match any Pfam domain, a percentage that reached 80% for the small secreted proteins, indicating rapid evolution. In comparison to background genes, we indeed found SPs to be more differentiated among species and strains, more often under positive selection, and highly expressed *in planta*; repeat-induced point mutations (RIPs) had no role in effector diversification, as SPs were not closer to transposable elements than background genes and were not more RIP affected. Our study thus identified both conserved core proteins, likely required for the pathogenic life cycle of all *Microbotryum* species, and proteins that were species specific or evolving under positive selection; these proteins may be involved in host specialization and/or coevolution. Most changes among closely related host-specific pathogens, however, involved rapid changes in sequences rather than gene gains/losses.

## Abstract (precis)

Plant pathogens use molecular weapons to successfully infect their hosts, secreting a large portfolio of various proteins and enzymes. Different plant species are often parasitized by host-specific pathogens; however, it is still unclear whether the molecular basis of such host specialization involves species-specific weapons or different variants of the same weapons. We therefore compared the genes encoding secreted proteins in three plant-castrating pathogens parasitizing different host plants, producing their spores in plant anthers by replacing pollen. We validated our predictions for secretion signals for some genes and checked that our predicted secreted proteins were often highly expressed during plant infection. While we found few species-specific secreted proteins, numerous genes encoding secreted proteins showed signs of rapid evolution and of natural selection. Our study thus found that most changes among closely related host-specific pathogens involved rapid adaptive changes in shared molecular weapons rather than innovations for new weapons.

## INTRODUCTION

Host specialization is a phenomenon well documented in many fungal pathogen/plant host systems (1), which most often occurs through host shifts (2). The ability to infect a new host is determined by the protein-protein interactions that occur at the pathogen-host interphase. For pathogens to be successful, they not only must be able to colonize the host but also must work around a gauntlet of host defense responses, as well as manipulate the host to their advantage. Pathogens accomplish these ends through the deployment of many secreted effectors (3–5).

It has been understood for several decades that plant pathogens utilize secreted effectors to infect their hosts (1, 6), including the maize pathogen member of the smut fungi Ustilago maydis (3). To defend against these pathogens, plants continuously evolve to recognize pathogen-associated molecular patterns and trigger a variety of immune responses (7). Reciprocally, there is an ongoing selective pressure for plant pathogens to adapt to their host by developing new effectors or otherwise alter the compositions of their secretomes to evade detection and find new ways to manipulate the host to their advantage. Secretomes can thus evolve rapidly, not only during host shift events but also due to intraspecific coevolution (8). It is, however, still unclear whether changes in secretomes leading to host specialization and local adaptation primarily involve effector gene gains/losses or changes in their sequences. A repeat-induced point mutation (RIP) is a fungal defense mechanism against transposable elements that has been suggested to play a role in effector diversification in fungi harboring effectors in regions rich in repetitive elements (9, 10). RIPs indeed act via mutations of repeated sequences at specific target sites and can “leak” on neighbor genes (9, 10).

Host specialization following host shift is particularly common in the fungal pathogen species complex *Microbotryum violaceum* (11). *Microbotryum* species are basidiomycete smut fungi that complete their life cycle in the anthers of their respective host plants, replacing the pollen with their own fungal spores (12). Originally described as a single species, these anther smuts are now understood to represent a complex of species (13, 14), most being highly specific to particular species of the Caryophyllaceae family, also known as “pinks” (15). Intraspecific coevolution has also been suggested to occur based on local adaptation patterns, where host plants were more resistant to their local sympatric anther smut pathogen than to those from geographically distant populations of the same species (16, 17).

To infect their hosts, *Microbotryum* fungi, like many other plant pathogens, employ an array of effector proteins to block plant immune response and otherwise manipulate the host during infection (18, 19). While the specificities of the various *Microbotryum* species to their corresponding host plants have been extensively described (14, 15, 20), the molecular basis for host specialization and coevolution within the complex has just recently begun to be explored (21–23). Understanding the changes that have occurred in the secretomes of these host-specific species will broaden our understanding of the mechanisms behind coevolution, host shifts, and emergent diseases. Furthermore, *Microbotryum* species offer a unique model system to study host shifts and specialization, with multiple host-specific and closely related pathogens (24), which is not often the case in agriculturally propagated crops.

To test whether host-specific or locally adapted closely related pathogens differed in their secretomes mainly by gene gains/losses or by rapid evolution of shared effectors, we compared the secretomes of three *Microbotryum* species, two sister species, *M. lychnidis-dioicae* and *M. silenes-dioicae*, and a more distantly related species, *M. violaceum* var. *paradoxa*. We sought to identify sets of secreted core proteins (i.e., orthologous genes encoding secreted proteins shared by all species) that likely play a major role in the pathogenicity of the species complex as a whole. We also sought to identify species-specific effectors and effectors evolving under positive selection and highly expressed *in planta*, thus perhaps involved in host specificity. To further our understanding of coevolution and local adaptation, we compared the secretomes of two *M. lychnidis-dioicae* strains collected from geographically distant populations belonging to distinct genetic clusters that have shown contrasted infection patterns consistent with local adaptation of plants (17). We also investigated whether the most frequent changes among host-specific species or locally adapted clusters involved mostly the gain/loss of secreted proteins or the diversification of shared proteins. As RIP-like footprints have been detected in *Microbotryum* fungi (25), we also tested whether sequence divergence in genes under positive selection and/or in genes encoding secreted proteins may have been facilitated by RIPs.

## RESULTS

### Overview of predicted *Microbotryum* secretomes.

**FIG 1:**
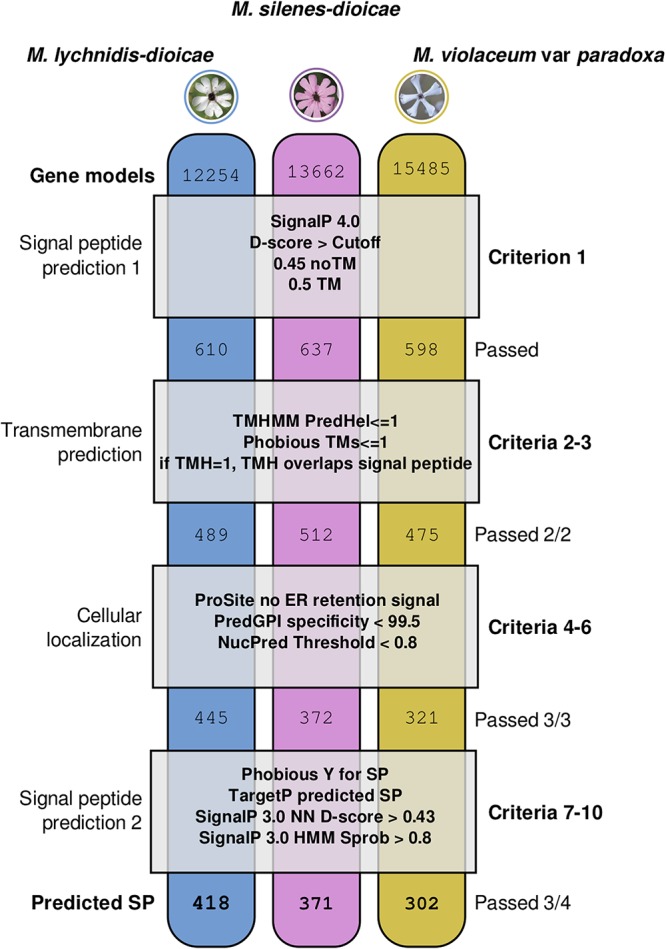
Procedural framework for predicting secreted proteins in three *Microbotryum* species. The genomes for the three fungal species (*M. lychnidis-dioicae*, *M. silenes-dioicae*, and *M. violaceum* var. *paradoxa*) were first screened to identify putative secreted proteins (SPs) (criterion 1). The resulting proteins were then screened for transmembrane (TM) segments (criteria 2 to 3) and for conflicting cellular localizations (criteria 4 to 6). Candidate secretory peptides were retained for further analysis if they passed all of the first six criteria (criteria 1 to 6) plus at least three out of four additional signal peptide prediction cutoffs (criteria 7 to 10). Each column corresponds to a species, each box corresponds to the criteria employed, and the numbers correspond to the translated gene models that passed the criteria above. PredHel, number of predicted transmembrane helices by N-best; TMH, number of predicted transmembrane helices; ER, endoplasmic reticulum; PredGPI, prediction of glycosylphosphatidylinositol anchored; NucPred, prediction of nuclear localization; Y, yes (predicted to be secreted by Phobius); Sprob, SignalP HMM secretion probability.

Analysis of the three *Microbotryum* secretomes revealed inventories of SPs of similar sizes in all three species. Initial prediction identified around 600 genes with signal peptides in each species (Fig. 1). Utilizing sequence-based criteria of cellular localization and secretory signals, we kept 302, 371, and 418 SPs in *M. violaceum* var. *paradoxa*, *M. silenes-dioicae*, and *M. lychnidis-dioicae*, respectively, for further analysis.

**FIG 2:**
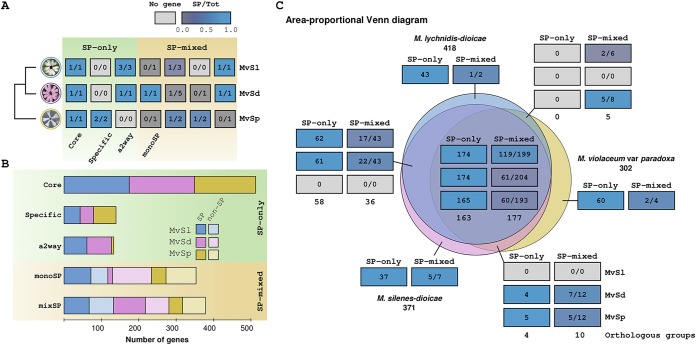
Comparison between the secretomes from three *Microbotryum* species. (A) Key to the phylogenetic profile of predicted SP and non-SP homologs, with examples for the orthologous group terminology used in this study. The cladogram on the left shows the phylogenetic relationships of the three species. In the SP-only orthologous groups (with the light-green background) at the left, all genes are predicted as secreted. In the core secretome, all three species have at least one predicted SP. In the species-specific orthologous groups, predicted SPs are represented in a single species (i.e., paralogous genes). In the accessory two-way (a2way) groups, one species did not have any ortholog in our reconstruction. In the SP-mixed orthologous groups (with the yellow background) at the right, not all orthologs were predicted as secreted; for example, in the monoSP group, a single species had predicted secreted proteins in the mono-copy orthologous group. The box color key corresponds to the ratio of predicted SPs over the total (Tot) number of genes in a given orthologous group per species, with a gradient from blue, when all orthologs in all three species are predicted as secreted, to dark gray, when no ortholog is predicted as secreted. Pale-gray boxes represent missing genes in a given orthologous group. (B) Stacked bar plots of gene counts in the different categories described in panel A, with the same terminology; light colors correspond to non-SP homologs of predicted SPs. (C) Area-proportional Venn diagram of predicted SP and non-SP homologs, as well as species-specific genes. Each area is annotated with six cell blocks, with the number/proportion of predicted SPs in the SP-only and SP-mixed orthologous groups, respectively, colored according to the same gradient as in panel A. Numbers at the bottom of the blocks correspond to the number of SP-only (left) or SP-mixed (right) orthologous groups. Rows in the blocks correspond to *M. lychnidis-dioicae*, *M. silenes-dioicae*, and *M. violaceum* var. *paradoxa*, from top to bottom. The Venn diagram was obtained with BioVenn (72). Abbreviations: a2way, accessory SP two-way orthologous groups; Core, orthologous groups in which all members are predicted as SPs and with at least one gene in each species; mixSP, orthologous groups with both SP and non-SP genes, not including monoSP; monoSP, orthologous groups with one gene in each species but with a single predicted SP; MvSl, *M. lychnidis-dioicae*; MvSd, *M. silenes-dioicae*; MvSp *M. violaceum* var. *paradoxa*; SP-mixed, orthologous groups with at least one gene not predicted as encoding an SP; SP-only, orthologous groups in which all genes are predicted as encoding SPs.

Over 85% of the predicted SPs were clustered into 453 orthologous groups, 225 comprising exclusively predicted SPs (645 SPs), henceforth called the SP-only group, and 239 in which at least one member was not predicted as an SP (298 SPs), henceforth called the SP-mixed group (Fig. 2). Over two-thirds of the predicted SPs belonged to orthologous groups with genes in all three species (753 predicted SPs in the 163 SP-only and 177 SP-mixed groups). Further, 190 predicted SPs belonged to orthologous groups shared by only two species. Only 148 SPs (i.e., 14% of the total) had no ortholog in two of the species and were therefore classified as species-specific SPs (62 in *M. violaceum* var. *paradoxa*, 44 in *M. lychnidis-dioicae*, and 42 in *M. silenes-dioicae*). Predicted SPs were significantly depleted in species-specific genes in all three species (chi-square test with Yates correction, *P* ≤ 0.0002). We classified as the “core secretome” 47% of the predicted SPs (513 genes belonging to 163 SP-only orthologous groups with members in all three species). In 118 SP-mixed orthologous groups with single-copy members in all three species, secretion signals were predicted in the orthologs of a single species, orthologs being non-SPs in the two other species; such orthologous groups are referred to as “monoSPs” here (Fig. 2 and Data Set S1 in the supplemental material).

10.1128/mBio.02391-19.2 DATA SET S1 Full annotation of predicted gene models for three *Microbotryum* species. Columns: 1, gene ID; 2, predicted SP (SPr1) or non-SP; 3, orthologous group ID (“xxAg” followed by a designation indicates that a gene model was not clustered into an orthologous group); 4, annotation class (AnnotR1); 5, protein length; 6, signal peptide length (lengthSP); 7, average *dN/dS* ratio; 8, positive selection (PS) (YES/NO); 9, best Pfam hit code; 10, distance to the nearest transposable element (distTE); 11, RIP index. Download Data Set S1, XLSX file, 2.3 MB. Copyright © 2019 Beckerson et al. 2019 Beckerson et al. This content is distributed under the terms of the Creative Commons Attribution 4.0 International license.

**FIG 3:**
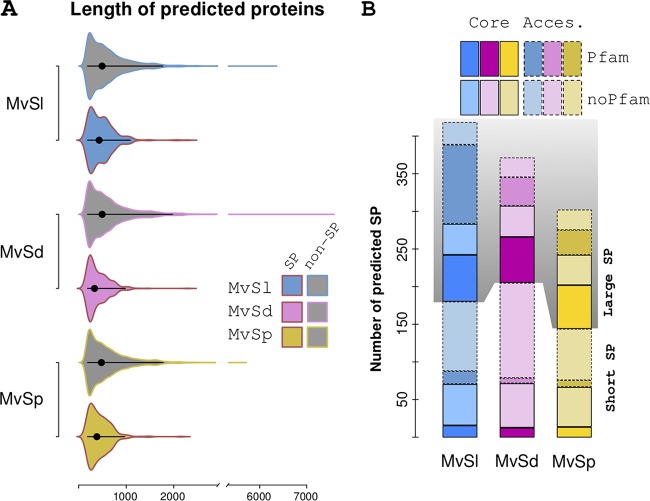
Overview of predicted SP (secreted protein) and non-SP homologs. (A) Length distribution of predicted SPs (area colored by species) and non-SPs (gray area with outline colored by species) in the three species. Black bars and large black dots indicate the range containing 95% of the points and the median, respectively. (B) Pfam screening results for predicted SPs in each of the three species. Stacked bars show the number of predicted SPs with (dark colors) and without (light colors) hits among Pfam-A models. Predicted SPs from the core secretome are boxed with a continuous line, and those from the accessory (Acces.) secretome are boxed with broken lines. The shaded area corresponds to predicted SPs larger than 250 amino acids (Large SP). *Microbotryum* species abbreviations are as defined in the legend of Fig. 2.

The majority of SPs for each species were smaller than the median length of all predicted proteins in the three species (57%, 68%, and 65% of SPs were smaller than 361 amino acids for *M. lychnidis-dioicae*, *M. silenes-dioicae*, and *M. violaceum* var. *paradoxa*, respectively) (Fig. 3A and Data Set S1). Initial screening of secretomes showed high percentages of SPs without known Pfam domains, i.e., 52.1% in *M. lychnidis-dioicae*, 67.9% in *M. silenes-dioicae*, and 62.3% in *M. violaceum* var. *paradoxa*. The percentages of genes without identified Pfam domains were even higher for predicted SPs smaller than 250 amino acids, i.e., 81.7% in *M. lychnidis-dioicae*, 88.9% in *M. silenes-dioicae*, and 84.0% in *M. violaceum* var. *paradoxa* (Fig. 3B). This trend was further observed when we analyzed the subset of core SPs (Fig. 3 and Data Set S1).

### Signal peptide clusters and yeast secretion trap results.

**FIG 4:**
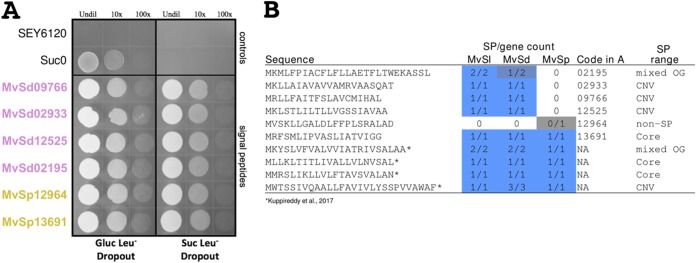
Experimental validation of predicted signal peptides. (A) Yeast secretion trap analysis of a subset of putative secreted proteins from *Microbotryum silenes-dioicae* and *M. violaceum* var. *paradoxa.* The invertase-deficient mutant SEY6120 of Saccharomyces cerevisiae is shown in the top row and represents a negative control on medium containing sucrose as the sole carbon source. SEY6120 cells transformed with the pYST-0 vector without a signal peptide upstream of the invertase gene is shown in the second row. Such cells are able to grow on the glucose –Leu dropout medium but not when sucrose is the sole carbon source. The SEY6120 cells in the subsequent six rows are transformed with a construct in which the signal peptide region corresponding to the putative secreted protein ID listed on the left of the row is fused to the truncated *SUC2* gene. If the signal peptide allows secretion, then the transformed S. cerevisiae cells are able to grow on sucrose as the sole carbon source. Different dilutions of cells were made (undiluted [Undil], diluted 10×, or diluted 100×) to better distinguish differences, if any. (B) Amino acid sequences and species ranges of signal peptides tested here and in a previous study (19). Cells under the “SP/gene count” columns follow the same color scheme as in Fig. 2. *Microbotryum* species abbreviations are as defined in the legend of Fig. 2. The signal peptide with the code 12964 in panel A corresponds to a protein from *M. violaceum* var. *paradoxa* predicted to be GPI anchored to the membrane. NA, not applicable; OG, orthologous groups; CNV, copy number variation.

The clustering of the signal peptides of predicted SPs resulted in 280 groups with two or more sequences at 75% sequence identity (823 sequences out of the 1,091 predicted SPs). The signal peptides tested here together with the four previously tested (19) are representative of the signal peptides of 28 predicted SPs in the three *Microbotryum* species under study (Fig. 4). To test whether the predicted secretion signals can indeed direct secretion, we used an invertase-deficient mutant of Saccharomyces cerevisiae. Such mutants can grow on glucose but not on sucrose unless transformed with a plasmid containing the invertase gene with a functional secretion signal, which allows the invertase to cleave extracellular sucrose into glucose and fructose in the medium. Cells of the invertase-deficient mutant SEY6120 of S. cerevisiae were transformed with pYST-0 vectors containing each tested signal peptide region upstream and in frame with the invertase gene. As evidenced by the ability of their respective secretion signals to allow SEY6120 to grow on medium containing sucrose as the sole carbon source, all 9 predicted secreted proteins that have been tested so far using a yeast secretion trap have been confirmed to be secreted (Fig. 4 and see reference 19). Interestingly, protein 12964 from *M. violaceum* var. *paradoxa* was originally filtered out of our list of predicted SPs, due to the prediction that it is anchored with glycosylphosphatidylinositol (GPI) to the membrane. Nevertheless, in this assay using only the secretion signal of the protein, invertase was secreted, suggesting that our conservative approach for estimating secretion may initially filter out membrane proteins with potential functional components outside the fungal cell.

### Interspecies comparison of *Microbotryum*-predicted secretomes.

**FIG 5:**
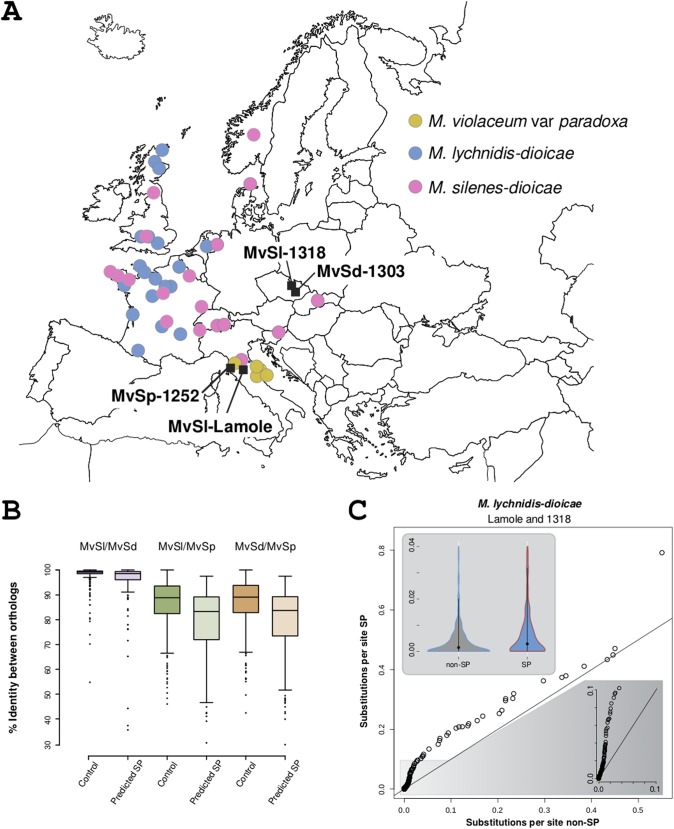
Inter- and intraspecific comparisons of *Microbotryum* secretomes. (A) Sampling locations of the isolates used in this study. (B) Distribution of pairwise percentages of amino acid sequence identity between predicted SPs and background orthologous genes from *M. lychnidis-dioicae*, *M. silenes-dioicae*, and *M. violaceum* var. *paradoxa*. (C) Quantile-quantile (main) and violin (inset) plots of substitution numbers per site between two strains of *M. lychnidis-dioicae* from Lamole, Italy (MvSl-Lamole), and from Olomouc, Czech Republic (MvSl-1318). The shaded area at the bottom right zooms into the low-divergence zone of the quantile-quantile plot. The straight lines correspond to a 45-degree reference line (i.e., points would fall close to this line if the two data sets have the same distribution). *Microbotryum* species abbreviations in panels A and B are as defined in the legend of Fig. 2.

As expected due to their phylogenetic placement, the orthologous proteins of *M. silenes-dioicae* and *M. lychnidis-dioicae* were more similar (median identity, 98.7%) than either of the two sister groups to *M. violaceum* var. *paradoxa* (medians, 86.9% for *M. lychnidis-dioicae*/*M. violaceum* var. *paradoxa* and 87.1% for *M. silenes-dioicae*/*M. violaceum* var. *paradoxa*). Orthologous SPs, including those belonging to the core secretome, were significantly less similar to one another than control non-SPs from single-copy orthologous groups of similar lengths (Wilcoxon rank sum test with continuity correction, *P* < 7e–7 for all three pairwise between-species comparisons) (Fig. 5). Out of the 150 single-copy orthologous groups with an SP predicted in each of the three species, i.e., most of what we call the core secretome (leaving out 13 single-copy orthologous groups with more than one gene in at least one species), we identified 92 groups with codons exhibiting more nonsynonymous substitutions than synonymous substitutions. Likelihood ratio tests comparing models with and without positive selection indicated that the model with positive selection was significantly more likely in 18 of these groups (Bonferroni multiple-test-corrected *P* value, <0.05) (Data Set S2). Similarly, we identified 74 out of 118 monoSP orthologous groups with codons exhibiting ratios of nonsynonymous to synonymous evolutionary changes (*dN/dS* ratios) above 1, among which multiple-test-corrected likelihood ratio tests revealed 21 orthologous groups evolving under positive selection. Selection tests of the 314 control orthologous groups with lengths similar to those of SPs returned 20 groups evolving under positive selection. Core secretome and monoSP orthologous groups were found enriched in proteins with signs of positive selection (Fisher’s exact text, *P* = 0.02505 for core versus control and *P* < 0.00048 for monoSP versus control) (Data Sets S1 and S2). We found 9 core and 14 monoSP orthologous groups under positive selection with hits in the Pfam-A database (Data Set S1), among which pectinesterase (PF01095.19) and chitin deacetylase (PF01522.21) have been implicated in fungal biotrophy, potentially for the manipulation of host development (18, 26). Glycosyl hydrolases (GHs) (PF00295.17 and PF00704.28) were found in the core and monoSP orthologous groups, despite an overall paucity of GHs represented among *M. lychnidis-dioicae* genes (18). Enzymes of these particular families are interesting due to their ability to hydrolyze pectin, a process important in both pathogenic- and saprophytic-fungus life stages (27).

10.1128/mBio.02391-19.3 DATA SET S2 Interspecific selection tests (Selecton) on three *Microbotryum* species. Columns: 1, orthologous group codes (Agogue); 2, annotation classes (monoR1, coreR1, contR1); 3, log likelihood M8; 4, log likelihood M8a; 5, average *dN/dS* ratios (AVGdN/dS); 6, likelihood ratio test (LRT) results; 7, Bonferroni-adjusted *P* value (p.adj); 8, positive selection (PS) (YES or NO). Download Data Set S2, TXT file, 0.03 MB. Copyright © 2019 Beckerson et al. 2019 Beckerson et al. This content is distributed under the terms of the Creative Commons Attribution 4.0 International license.

### Intraspecific comparisons of *Microbotryum-*predicted secretomes.

We further investigated footprints of positive selection using McDonald-Kreitman (MK) tests that compare the amount of variation within a species (polymorphism) to the divergence between species (substitutions) at two types of sites, synonymous and nonsynonymous. A ratio of nonsynonymous to synonymous polymorphisms within species lower than the ratio of nonsynonymous to synonymous differences between species indicates positive selection (28). We performed three pairwise species comparisons between *M. violaceum* var. *paradoxa*, *M. lychnidis-dioicae*, and *M. silenes-dioicae*, using 148 core, 115 monoSP, and 314 control orthologous groups. We used population genomics data from 20, 18, and 4 isolates from *M. lychnidis-dioicae*, *M. silenes-dioicae*, and *M. violaceum* var. *paradoxa*, respectively (22, 29, 30) (Table S1). Figure 5A shows the locations where the isolates were sampled. The MK tests indicated signatures of within-species positive selection in 8 core secretome orthologous groups and 15 monoSP orthologous groups (Data Set S3). Out of the 23 orthologous groups with signatures of positive selection detected using MK tests, 6 were also detected to evolve under positive selection in the Selecton analysis (Data Set S1). Five orthologous groups were found undergoing intraspecific positive selection in all three comparisons. Intraspecific selection tests on control non-SP orthologous groups revealed that 11 underwent positive selection. While core SPs showed no excess of fixed nonsynonymous polymorphisms, monoSPs were enriched in genes evolving under within-species positive selection (15 out of 115 monoSPs versus 11 out of 314 non-SP genes; Fisher’s exact test, *P* = 0.0008147).

10.1128/mBio.02391-19.1 TABLE S1 Isolates of *Microbotryum* species and accession numbers of population genomics data. Columns: A, sample ID; B, fungal species; C, host species; D, BioProject ID; E, Sequence Read Archive accession number. Download Table S1, XLSX file, 0.01 MB. Copyright © 2019 Beckerson et al. 2019 Beckerson et al. This content is distributed under the terms of the Creative Commons Attribution 4.0 International license.

10.1128/mBio.02391-19.4 DATA SET S3 Intraspecific selection tests (MK test) results with three *Microbotryum* species. Columns: 1, orthologous group code (Agogue); 2, annotation class (AnnotR1); 3, predicted SPs (rangeP) in MvSl (100), MvSd (010), MvSp (001), all three species (111), or none of the species (000); 4, MK test results; 5, nonsynonymous polymorphisms in population 1 (P1_nonsyn); 6, nonsynonymous polymorphisms in population 2 (P2_nonsyn); 7, synonymous polymorphisms in population 1 (P1_syn); 8, synonymous polymorphisms in population 2 (P2_syn); 9, nonsynonymous substitutions between species 1 and 2 (D_nonsyn); 10, synonymous substitutions between species 1 and 2 (D_syn); 11, neutrality index; 12, alpha parameter (alpha); and 13, Fisher’s adjusted *P* value. Download Data Set S3, TXT file, 0.1 MB. Copyright © 2019 Beckerson et al. 2019 Beckerson et al. This content is distributed under the terms of the Creative Commons Attribution 4.0 International license.

When we compared two well-assembled *M. lychnidis-dioicae* genomes, those of the Lamole and 1318 strains, originating from two differentiated populations maladapted to their sympatric hosts (17), we found only 29 Lamole *M. lychnidis-dioicae* SPs without a corresponding 1318 *M. lychnidis-dioicae* gene (12 predicted SPs in 10 orthologous groups and 17 species- or strain-specific SPs). In addition, we found 11 orthologous groups for which gene model counts were different between the 1318 and Lamole *M. lychnidis-dioicae* strains. The ratio of SP-containing orthologous groups with gene count polymorphisms between *M. lychnidis-dioicae* strains was significantly smaller than the genome-wide ratio (21/357 SPs versus 2,642/12,277 total genes; chi-square with Yates correction, *P* < 1e–11). We found few predicted SPs within genome regions showing a presence/absence polymorphism within species, as analyzed previously (21), in both *M. lychnidis-dioicae* Lamole (five) and *M. silenes-dioicae* (two). Substitutions in predicted SPs, on the other hand, were more frequent between *M. lychnidis-dioicae* Lamole and *M. lychnidis-dioicae* 1318 strains than substitutions in control genes (Wilcox rank sum test with continuity correction, *P* = 2.537e–05) (Fig. 5C and Data Set S4).

10.1128/mBio.02391-19.5 DATA SET S4 Per-gene substitutions between *Microbotryum lychnidis-dioicae* strains Lamole and 1318. Columns: 1, orthologous group code (Agogue); 2, annotation class (AnnotR1); 3, codon alignment length (alnL); 4, nonsynonymous polymorphisms (PN); 5, synonymous polymorphisms (PS); 6, absolute distance (Pdist) [(PN + PS)/alnL]. Download Data Set S4, TXT file, 0.02 MB. Copyright © 2019 Beckerson et al. 2019 Beckerson et al. This content is distributed under the terms of the Creative Commons Attribution 4.0 International license.

### Genomic context of predicted SPs.

**FIG 6:**
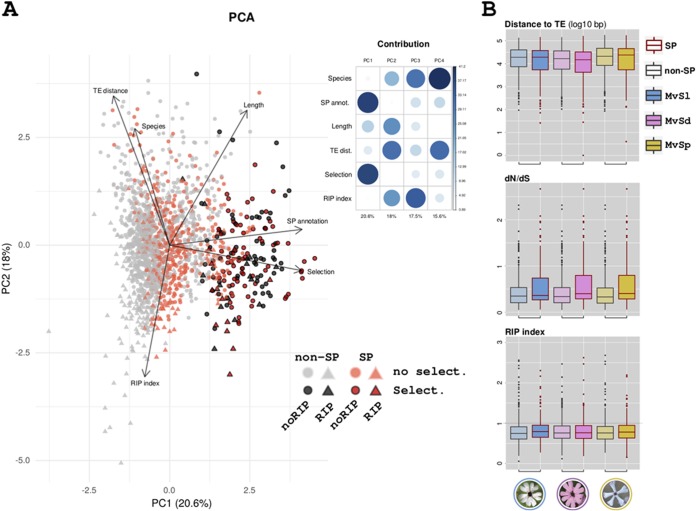
Investigation of the impact of RIPs (repeat-induced point mutations) on gene diversification among species. (A) Principal-component analysis (PCA) of gene copies according to their trait values for six variables: (i) their annotation (annot.) as a binary variable, i.e., encoding SPs (genes colored in red) or non-SPs (in gray); (ii) their length in base pairs as a continuous variable; (iii) the species to which they belong as a category variable (MvSl, *Microbotryum lychnidis-dioicae*; MvSd, *M. silenes-dioicae*; MvSp, *M. violaceum* var. *paradoxa*); (iv) their distance to the nearest transposable element as a continuous variable (TE dist.); (v) their RIP index as a continuous variable (RIP-affected genes are triangles, and non-RIP-affected genes are circles); and (vi) the detection of positive selection (genes with dark colors) or the lack of positive selection (light colors) as a binary variable. The projection of the variables is plotted as arrows in the space defined by the first (PC1) and second (PC2) components, and the percentage of the total variance explained by each principal component is provided in parentheses. The arrows representing the variable projections were scaled for better visualization (6-fold magnification). The contribution of the variables to principal components is shown in a correlation plot (upper right). no select., no selection. (B) TE distance, *dN/dS* ratio (synonymous substitutions over nonsynonymous substitutions), and RIP index distributions of predicted SPs (red contour) or non-SPs (gray contour) in the three species (areas are colored according to species). The distance to the TE was transformed as the log_10_ base pair distance, and the *dN/dS* ratio was calculated within orthologous groups. The boxplots represent the median (center line), the 25th percentile, the 75th percentile (box boundaries), and 1.5 times the distance between the 25th and the 75th percentiles (whiskers); points are the outliers.

Unlike with some other plant-pathogenic fungi with effectors frequently located in repeat-rich regions, we did not find genes encoding predicted SPs to be significantly closer to transposable elements than other genes (Fig. 6) and found no evidence for genome compartmentalization into AT-rich or GC-rich regions in any of the three genomes analyzed, extending previous observations (18). We nevertheless estimated the frequency of sites potentially affected by the RIP-like mechanism reported in *Microbotryum* fungi, targeting TTG and CAA trinucleotides. We calculated an RIP index that takes values above 1 when there is an excess of TTG and CAA trinucleotides over the corresponding target sites not affected by RIP (TCG and CGA), controlling for local sequence composition (see Materials and Methods). The coding regions of predicted SPs did not show any significant excess of RIP-affected trinucleotides, regardless of whether the orthologous groups showed signs of positive selection (Fig. 6). Our RIP index measure was negatively correlated with the distance to transposable elements (TEs), indicating RIP leakage to TE-neighboring regions. The RIP index was not correlated with the ratio of nonsynonymous to synonymous substitutions (Fig. 6), indicating that the RIP-like mechanism does not play a significant role in the diversification of genes under positive selection in *Microbotryum* fungi.

### Expression of predicted SPs across infection stages.

**FIG 7:**
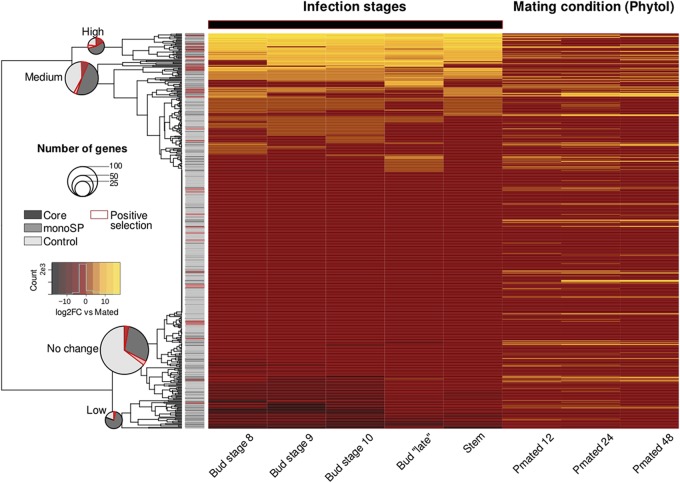
Relative expression of *Microbotryum lychnidis-dioicae* genes across infection stages on flower structures. Heatmap of average gene expression (*n* = 2 to 4) across infection stages in flower structures (32) and under mating conditions (31) as a log_2_ fold change against a noninfection condition (mating on phytol, “Pmated”). Hierarchical clustering based on mean row values across the infection stages (horizontal black bar) distinguish four expression profiles with average log_2_ fold change median values as follows: low, −6; no change, 0; medium, 1.36; and high, 12. The sidebar represents the annotation of the genes according to the color scheme on the left. Pie charts detail the proportions of SP (core and monoSP) and non-SP (control) genes in each expression profile cluster. The pie chart area is proportional to the number of genes in each expression profile cluster. Red shades and outlines indicate genes with signatures of positive selection.

We focused our analysis on *M. lychnidis-dioicae* Lamole genes expressed in at least one of the five infection stages or under three mating conditions for which we retrieved expression data (18, 31, 32). Among the 2,840 genes fulfilling this condition, we found 135 and 58 predicted SPs from the single-copy core and monoSP orthologous groups, respectively, and compared their expression profiles to those of 232 genes from the non-SP control group (same length distribution but not predicted as potential effectors). Hierarchical clustering of expression profiles across infection stages grouped the genes into low (31 genes, median log_2_ fold change [FC] range, −7.35 to 4.15), medium (117 genes, median log_2_ FC range, 0.0 to 1.8), high (29 genes, median log_2_ FC range, 9.19 to 12.40), and no-change (248 genes, median log_2_ FC, 0) average gene expression profiles across infection stages. We found no major changes in the expression of core, monoSP, or non-SP genes across three mating conditions. Predicted SPs from the core orthologous groups were enriched among genes with high or low levels of average expression across infection stages, respectively (19 and 18 out of 135 core SPs compared with 7 and 6 out of 232 control genes; Fisher’s two-tailed exact test, *P* = 1.8E–3 and 1.1E–3, respectively) (Fig. 7). In line with the pattern observed across all predicted SPs, we could infer the functions of only 14 core and 7 monoSP genes with either high or low average expression. Glycosyl hydrolases, often involved in pathogenesis (27), were among the most common hits (Data Sets S1 and S5).

## DISCUSSION

*Microbotryum* secretomes appeared as largely shared among species, i.e., with few gene gains/losses. Instead, we found SPs to be rapidly evolving, as these were more differentiated among species and more often under positive selection than non-SP genes, indicating that many SPs likely evolved under diversifying selection among species parasitizing different hosts. Such rapid evolution was also indicated by the low percentage of SPs matching Pfam domains (31 to 47%), a percentage that decreased to less than 20% for the small secreted proteins. Such a finding regarding the lack of identifiable Pfam domains of a substantial proportion of SPs is consistent with previous reports of other smut pathogens and is a hallmark of secreted effectors involved in host specificity (33). Diversifying selection in *Microbotryum* SPs is likely due to coevolution within species, local adaptation, or specialization to different hosts, involving rapid changes in the sequences of secreted proteins to avoid detection in the plant and, more generally, to counteract evolving host defenses. Such a hypothesis is reinforced by the finding that SPs under positive selection were more often highly expressed *in planta* than non-SP genes. Although we found few species-specific SPs or SPs with copy number variation, these accessory SPs may also be involved in coevolution, local adaptation, and/or host specialization (34, 35).

The results from the intraspecific comparison between the two *M. lychnidis-dioicae* strains shed further light on coevolution and local adaptation. We indeed found SPs to be more differentiated than non-SPs between two strains from genetically differentiated populations. These findings further support the idea that coevolutionary pressures may cause divergence in effectors between differentiated populations of pathogens. In fact, the populations from southern and eastern Europe were genetically differentiated in both *M. lychnidis-dioicae* and its host plant *Silene latifolia*, and the plant showed local adaptation to the fungus (17), indicating the occurrence of coevolution. Gene presence/absence polymorphisms in *M. lychnidis-dioicae*, corresponding to the pathogen and host phylogeographic structure (21), and numerous selective sweeps across the genome (22) further supported the existence of coevolution. Unlike with several crop pathogens (36, 37), neither presence/absence polymorphisms nor selective sweep regions were enriched in predicted SPs, even though nearly 10% of SPs were found located within recent selective sweeps of *M. lychnidis-dioicae*, which suggests recent adaptive events involving some SPs.

The identification of a set of shared and conserved SPs, i.e., the 126 core secretome orthologous groups without positive selection, was also interesting, providing a starting point to search for effectors that play a central role in the common pathogenicity traits of these fungi, e.g., the effectors that allow the fungi to migrate to the plant anthers, to induce stunted ovary and pseudoanther development in female flowers, and to eliminate and replace host pollen with fungal spores. The observed differential expression of secreted core proteins further narrows the search for these central effectors and points to sets of genes within the secretome that may play other central roles in the fungal life cycle, including the secretion of extracellular enzymes for carbon source metabolism. Indeed, phosphatases, peptidases, lipases, and glycosidases accounted for half of the Pfam annotations of core secretome orthologous groups with no signs of positive selection (20 out of 38). While such enzymes are clearly associated with fungal pathogens (38–40), they are often found in animal (38, 39) and necrotrophic plant (27, 41, 42) pathogens, rather than in biotrophic fungi. On the other hand, the upregulation of many active carbohydrate enzyme genes related to cell wall degradation was also seen in both wheat stem and poplar rust, *Puccinia graminis* and *M. larici-populina*, respectively (43). In the case of *M. lychnidis-dioicae*, GH28 polygalacturonase domain-containing proteins were upregulated during infection and were among the proteins with signs of positive selection enriched in the core secretome and monoSP orthologous groups. Since polygalacturonase is required for the pathway implicated in pollen dehiscence (44), this is consistent with a fundamental role for such enzymes in the pathogenic lifestyles of anther smut fungi.

Future research with *Microbotryum* will utilize these findings to better understand the functions of the most promising SP candidates, by identifying their targets within each host. Such research geared toward identifying the targets of secreted effectors from *M. lychnidis-dioicae* in its corresponding host plant *Silene latifolia* has already made progress (19). For instance, we identified here MvSl-1064-A1-R4_MC02g04003 as part of the core secretome undergoing diversifying selection across species. We also found its transcript to be among the most highly expressed across infection stages. Its predicted protein product (residues 21 to 156) has been shown to interact with two host proteins in yeast two-hybrid assays (19). Extension of such work to analyze candidate effectors herein identified through *in silico* studies should add new insights into their relevance in host preference and the evolution of the *Microbotryum* species complex. By narrowing down the genomes and identifying prime candidates that are likely to play a major role in the pathogen’s life cycle, this work helps to bridge the gap between the quickly expanding availability of *Microbotryum* genomes (24, 30, 45) and the emerging cellular and molecular biology work being done to understand the role of effectors in this system (19).

More generally, this study showed that the molecular changes that lead to different host ranges between closely related plant pathogens, or between different locally adapted genetic clusters, involved few gene gains/losses in their secretome but instead rapid evolution of shared secreted proteins. This represents a significant advance in our understanding of pathogen evolution and may contribute to understanding host shifts and emergent diseases.

## MATERIALS AND METHODS

### Comparative genomics.

To analyze the relationship between various predicted effectors, we performed genomic analyses on the following available genomes, obtained using Pacific Bioscience (PacBio) single-molecule real-time sequencing: GCA_900015465.1 for *M. lychnidis-dioicae* Lamole a_1_ (Italy) (45), GCA_900015495.1 for *M. violaceum* var. *paradoxa* from *Silene paradoxa* 1252 a_1_ (30), and QPIF00000000 for *M. silenes-dioicae* 1303 a_2_ (45). These genomes were selected for comparison due to their relationship to one another; *M. lychnidis-dioicae* strains and *M. silenes-dioicae* are sister species, able to infect one another’s host in the greenhouse, although they do so to a lesser degree than their natural host (46) and very little in natural populations (47), while *M. violaceum* var. *paradoxa* serves as an outgroup, unable to infect either of the sister species’ hosts or vice versa (20).

In total, we used eight sequence-based prediction tools to identify potential effectors by searching each genome for genes with hallmarks of secretion and without conflicting cellular localization predictions. The initial list of putative secreted proteins (SPs) were generated by running the entire genomes through SignalP 4.0 (48). In order to increase the stringency of this analysis, the SPs must then have passed the following criteria to rule out potential localization or retention in various membranes within or on the cell, which is similar to the previously published protocol for *M. lychnidis-dioicae* (18). Potential transmembrane domains were predicted with TMHMM (49) and Phobius (50). Only gene models with none or a single transmembrane domain prediction overlapping the signal peptide prediction were considered further (18, 48). Prosite was used to screen for predicted endoplasmic reticulum retention signals, while PredGPI (51) was used to screen for potential glycosylphosphatidylinositol anchors, and NucPred (52) was used to screen for nuclear localization signals in the predicted protein (Fig. 1).

Gene models predicted to be secreted and without conflicting localization predictions (i.e., negative for transmembrane domains, endoplasmic reticulum retention, GPI anchoring, and nuclear localization) were further screened using additional criteria to identify strong predictive footprints of secretion in the signal peptide region. To qualify as an SP, the candidates must also have passed stringent cutoff values for secretion, listed in Fig. 1, for at least three of the following four tests: a predicted secretion signal by TargetP (53), a D score (neural network predictors) of greater than 0.43 for the neural network (NN), a secretion probability of greater than 0.8 for the hidden Markov model (HMM) from SignalP3.0, and predicted secretion by Phobius.

We searched the resulting putative SPs among the orthologous groups reconstructed previously (30). Briefly, the orthologous groups were obtained using mcl (54) to cluster high-scoring blastp matches between all gene models predicted in 15 haploid genomes from eight *Microbotryum* species, previously parsed with orthAgogue (55). We classified a predicted SP as a species-specific SP if there was no ortholog in two of the species being considered. For predicted SPs belonging to orthologous groups, we distinguished between species-specific, two- or three-way orthologous groups (i.e., predicted as an SP in a single species or in two or three species, respectively) and between orthologous groups composed exclusively of predicted SPs (SP-only group members) and those containing at least one gene model not predicted as SP (SP-mixed group members). We defined the core secretome as the full set of predicted SPs belonging to SP-only three-way orthologous groups (i.e., present and predicted as SPs in all three species). Conversely, we defined the accessory secretome as the predicted SPs that were either species specific or belonged to SP-mixed or two-way SP-only orthologous groups (i.e., were not present in all species or not predicted as SPs in all species) (Fig. 2). Together, the core and accessory secretomes make up the pan-secretome, i.e., the full set of predicted SPs in all species considered.

### Pfam domain annotation.

We searched Pfam release 32 (56) against the translated gene models of all predicted SPs and their homologs with hmmsearch from the HMMER 3.1b1 suite (http://hmmer.org). Hits with an E value smaller than 1e–3 were considered significant. The results were then categorized by size as well as presence/absence of a predicted Pfam domain (see Data Set S1 in the supplemental material).

### Signal peptide clustering and experimental validation.

We clustered the predicted signal peptide sequences with CD-HIT (57), allowing for up to five amino acid differences (nondefault options, -c [sequence identity threshold] 0.75 -l [length of throw_away_sequences] 5). We tested whether predicted signal peptides could direct the secretion of the Suc2 invertase employing a yeast-based secretion trap method (19, 58). Six signal peptide-encoding sequences, as determined by SignalP 4.1 software, were amplified by PCR. A standard PCR cycle was used, with initial denaturation set at 94°C for 4 min and 35 cycles of 94°C for 30 s, 60°C for 30 s, and 72°C for 30 s and a final extension time of 5 min at 72°C. The purified fragments were then subcloned into a TOPO vector using an Invitrogen TOPO TA cloning kit and subjected to restriction digestion with EcoRI and NotI enzymes. The digested fragments were then purified and cloned into the pYST-0 vector, upstream and in frame with an invertase coding sequence, *SUC2*. The presence of each signal peptide encoded in frame with the *SUC2* coding region was confirmed by DNA sequencing (Eurofins, Louisville, KY).

Invertase-deficient (*suc2*-negative) Saccharomyces cerevisiae strain SEY 6210 (*MAT*α *leu2-3*,*112 ura3-52 his-*Δ2*00 trp1-*Δ*901 lys2-801 suc2-*Δ*9 GAL*) cells were transformed with the constructs using the Frozen-EZ yeast transformation II kit from Zymo Research. Cells were then suspended in water and spread onto dropout selection plates with synthetic defined medium (SD) lacking Leu (SD/–Leu) (Clontech) and either sucrose as the sole carbon source or glucose as a control. Resulting colonies from the sucrose plates were grown overnight in 3 ml of SD/–Leu broth with sucrose; 10-μl undiluted samples, 10-fold dilutions, and 100-fold dilutions were spotted onto SD/–Leu with glucose or sucrose as the carbon source and incubated for 2 days at 30°C. Clones harboring functional signal peptides with the reconstituted invertase activity were able to grow on sucrose as the sole carbon source. Untransformed mutant yeast strain SEY 6210 and transformed SEY 6210 cells with the empty pYST-0 vector were used as negative controls. Plasmid DNA was extracted from the positive clones and used to retransform Escherichia coli. The constructs were again checked for the presence of signal peptide sequence by DNA sequencing (Eurofins, Louisville, KY).

### Tests for positive selection.

We focused our selection analysis on single-copy three-way orthologous groups with one or three predicted SPs. We found 163 three-way SP-only orthologous groups, among which 150 were single-copy orthologous groups (i.e., single-copy three-way SP-only orthologous groups or single-copy core secretome). Furthermore, 118 single-copy orthologous groups retained a single predicted SP after annotation (i.e., single-copy three-way SP-mixed orthologous groups from the accessory secretome, here abbreviated monoSP). As a first method to test for positive selection, we compared evolutionary codon models M8 and M8a (59) on 150 core and 118 monoSP single-copy orthologous groups using Selecton (60). To check whether positive selection was more or less frequent in SP genes than in other (non-SP) genes, we performed the same test on 314 randomly picked single-copy three-way orthologous groups without predicted SPs and with the same length distribution as predicted SPs. The evolutionary model M8, in which a proportion of sites is drawn from a category with a *dN/dS* ratio greater than 1 (i.e., it allows for sites undergoing positive selection), was tested against M8a, in which no site is allowed to have a *dN/dS* ratio larger than 1 (i.e., it does not allow for positive selection), using a likelihood ratio test with 1 degree of freedom to determine the statistical probability that the genes evolve under positive selection (61). We adjusted chi-square *P* values using Bonferroni’s correction for multiple testing in R with 582 tests.

We also performed McDonald-Kreitman (MK) tests to infer the existence of positive selection (28). MK tests contrast levels of polymorphism and divergence to test for a departure from neutrality in terms of nonsynonymous substitutions (i.e., rapid amino acid changes) while controlling for gene-specific mutation rates. MK tests estimate α, the fraction of amino acid substitutions that were driven by positive selection. To analyze within-species polymorphism, we used genome sequences previously obtained with Illumina paired-end sequencing technology for populations of the three focal species, *M. lychnidis-dioicae*, *M. silenes-dioicae*, and *M. violaceum* var. *paradoxa* (22, 29, 30). We downloaded raw data publicly available from the NCBI Sequence Read Archive (SRA) under the BioProject accession numbers PRJNA295022, PRJNA269361 and PRJEB16741. Four major genetic clusters were identified in Europe in *M. lychnidis-dioicae* (22), and we considered only strains belonging to the largest cluster in northwestern Europe so that population subdivision does not bias selection inferences. A list of the isolates used in the analysis is presented in Table S1. We processed the raw genome data of 18 *M. silenes-dioicae*, 20 *M. lychnidis-dioicae*, and 4 *M. violaceum* var. *paradoxa* isolates to build pseudoalignment sequences of gene coding sequences within each species using as reference genomes the assemblies reported in GCA_900015465.1 for *M. lychnidis-dioicae*, GCA_900120095.1 for *M. silenes-dioicae*, and GCA_900015485.1 for *M. violaceum* var. *paradoxa*. First, reads were trimmed for quality (length, >50; quality base, >10) using the Cutadapt v1.12 software (62). We mapped Illumina reads against the reference genomes of each species using bowtie2 v2.1.0 (63) and filtered for PCR duplicates using Picard tools (http://broadinstitute.github.io/picard). We realigned reads, called for single nucleotide polymorphisms (SNPs), and filtered them for quality, high genotyping rate (>90%), and minor allele frequency (>10%) using GATK version 3.7 (64) and vcftools version 0.1.13 (65) as described previously (21, 30). We built pseudoalignment sequences of gene coding sequences from the VCF file produced by GATK using a customized script. For each strain, reference nucleotides were replaced by their variants in the reference sequence. We used MUSCLE (66) and TranslatorX (67) to perform codon-based alignments of gene coding sequences among and between species. We used the MKT() and get.MKT() functions in the PopGenome R package (68) to perform MK tests.

With these tools, we performed three comparisons. We tested for positive selection comparing polymorphisms and the levels of divergence of 148 core secretomes and 115 monoSP orthologous groups for (i) *M. violaceum* var. *paradoxa* against *M. lychnidis-dioicae* and *M. silenes-dioicae* strains, (ii) *M. silenes-dioicae* against *M. violaceum* var. *paradoxa* strains, and (iii) *M. lychnidis-dioicae* against *M. violaceum* var. *paradoxa* strains. We excluded from the analyses genes having multiple (paralogous) copies. No neutrality index or *α* value could be computed for 27 orthologous groups in pairwise species comparison i, 67 orthologous groups in pairwise species comparison ii, and 67 orthologous groups in pairwise species comparison iii, due to a lack of synonymous or nonsynonymous polymorphisms. We performed the same three pairwise comparisons with 314 genes from the control group described above. No neutrality index or *α* value could be computed for 30, 99, and 84 orthologous groups in the control pairwise comparisons i, ii, and iii, respectively. We assessed the significance of positive selection for genes having a neutrality index inferior to 1 and a positive *α* value using a Fisher test (*P* value < 0.05).

### Footprints of RIPs.

We investigated the extent of RIP-like footprints in *Microbotryum* genomes with a per-gene repeat-induced point mutation (RIP) index defined as the ratio of *t* over *n* (*t/n*), with *t* being the sum of TTG and CAA trinucleotides (number of potentially RIP-affected forward and reverse targets, 24) divided by the sum of TCG and CGA trinucleotides (non-RIP-affected forward and reverse targets), with *n* being the sum of all other nontarget trinucleotides, [ACG]TG and CA[CGT], divided by the sum of [ACG]CG and CG[CGT], to control for contextual sequence composition. A RIP index greater than 1 thus represents an excess of potential RIP sites controlling for the base composition. We compared the distributions of per-gene RIP index values between genes predicted to encode SPs and those not predicted to encode SPs (non-SPs), considering whether or not the genes belonged to orthologous groups undergoing positive selection.

### Genomic landscape analyses.

We used OcculterCut v1.1 (69) to determine if *Microbotryum* genomes harbored AT-rich regions. Contigs suspected of containing mitochondrial sequences were removed from the assemblies prior to the analysis using the mito_filter.sh script, available as part of the OcculterCut distribution (https://sourceforge.net/projects/occultercut). Transposable element (TE) locations for *M. lychnidis-dioicae* and *M. silenes-dioicae* were retrieved from a previous study (21) and predicted in *M. violaceum* var. *paradoxa* using the same TE centroid sequence database (21). The distance to TE was parsed with bedtools (70).

### Intraspecific secretome comparison of *M. lychnidis-dioicae* isolates from differentiated populations.

For analyzing the genome-wide intraspecific variation in secretomes, a second genome (assembly GCA_003121365.1) of *M. lychnidis-dioicae* isolated in Olomouc, Czech Republic, and abbreviated as *M. lychnidis-dioicae* 1318, was analyzed (21). We used blastp and orthAgogue to obtain high-scoring pairs between gene models of *M. lychnidis-dioicae* 1318 and the entire gene model set analyzed previously (30) and reran the mcl algorithm. We then parsed the extended orthologous groups to identify the *M. lychnidis-dioicae* 1318 gene models homologous to the *M. lychnidis-dioicae* Lamole SPs identified in this work. We compared the frequencies of synonymous and nonsynonymous single nucleotide substitutions in codon-based pairwise alignments of *M. lychnidis-dioicae* Lamole and *M. lychnidis-dioicae* 1318 genes corresponding to the core secretome or to the non-SP control single-copy orthologous groups. Per-site substitution numbers were calculated as the sum of substitutions divided by the length of the nucleotide alignment.

### Analysis of gene expression level across infection stages and mating conditions.

We retrieved gene expression data across *M. lychnidis-dioicae* Lamole infection stages under *Silene latifolia* and phytol-induced mating conditions from previous studies (18, 31, 32) as the average log_2_ fold change (log_2_ FC) against the mated (noninfection) condition (2 to 4 experiments were performed for each of the eight conditions analyzed). We obtained the one-to-one gene model correspondences between long- and short-read sequencing-based assemblies of the same *M. lychnidis-dioicae* Lamole strain as best reciprocal hits with blastp. We focused our analysis on predicted SPs from the core and monoSP orthologs, using gene models from the control set described above for comparisons. Only genes with a Benjamini-Hochberg adjusted *P* value lower than 1e–5 under at least one condition were considered. Clustering and plotting were performed in R with the heatmap.2 function of the gplots package, using 10 bins for coloring the log_2_ FC values and clustering by mean values per row. Pie charts were generated with the pie function of R base.

### Plotting, statistical tests, and figures.

Unless otherwise stated, all plots and statistical tests were performed in R version 3.6.1 (71). The final layout of the figures was produced with Inkscape version 0.92.3.

### Data availability.

Raw data of Illumina paired-end sequencing for populations of the three focal species, *M. lychnidis-dioicae*, *M. silenes-dioicae*, and *M. violaceum* var. *paradoxa* (22, 29, 30), were downloaded from the NCBI Sequence Read Archive (SRA) under the BioProject accession numbers PRJNA295022, PRJNA269361, and PRJEB16741. A list of the isolates used in this analysis along with their accession numbers is presented in Table S1. All RNA sequence data for this study are available in NCBI under BioProject accession number PRJNA246470.

10.1128/mBio.02391-19.6 DATA SET S5 Normalized expression of *Microbotryum lychnidis-dioicae* Lamole genes across infection stages and mating conditions. Columns: 1, gene ID; 2 to 6, log_2_ FCs across infection stages (32); 7 to 11, Benjamini-Hochberg’s corrected *P* values (also known as false-discovery rates [FDR]) across infection stages; 12 to 14, log_2_ FCs across mating conditions (31); 15 to 17, Benjamini-Hochberg’s corrected *P* values across mating conditions. Download Data Set S5, TXT file, 0.7 MB. Copyright © 2019 Beckerson et al. 2019 Beckerson et al. This content is distributed under the terms of the Creative Commons Attribution 4.0 International license.
